# Graves’ Disease Is Associated with a Defective Expression of the Immune Regulatory Molecule Galectin-9 in Antigen-Presenting Dendritic Cells

**DOI:** 10.1371/journal.pone.0123938

**Published:** 2015-04-16

**Authors:** Susanna Leskela, Ana Serrano, Hortensia de la Fuente, Ana Rodríguez-Muñoz, Ana Ramos-Levi, Miguel Sampedro-Nuñez, Francisco Sánchez-Madrid, Roberto González-Amaro, Mónica Marazuela

**Affiliations:** 1 Department of Endocrinology, Hospital Universitario de la Princesa, Instituto de Investigación Sanitaria Princesa, Universidad Autónoma de Madrid, Madrid, Spain; 2 Department of Immunology, Hospital Universitario de la Princesa, Instituto de Investigación Sanitaria Princesa, Universidad Autónoma de Madrid, Centro Nacional de Investigaciones Cardiovasculares Carlos III, Madrid, Spain; 3 Department of Immunology, School of Medicine, UASLP, San Luis Potosí, SLP, México; University of Bergen, NORWAY

## Abstract

**Introduction:**

Patients with autoimmune thyroid disease (AITD) show defects in their immune-regulatory mechanisms. Herein we assessed the expression and function of galectin-1 and galectin-9 (Gal-1, Gal-9) in dendritic cells (DCs) from patients with AITD.

**Materials and Methods:**

Peripheral blood samples from 25 patients with Graves’ disease (GD), 11 Hashimoto’s thyroiditis (HT), and 24 healthy subjects were studied. Thyroid tissue samples from 44 patients with AITD and 22 patients with goiter were also analyzed. Expression and function of Gal-1 and Gal-9 was assessed by quantitative RT-PCR, immunofluorescence and flow cytometry.

**Results:**

A diminished expression of Gal-9, but not of Gal-1, by peripheral blood DCs was observed in GD patients, mainly in those with Graves´ ophthalmopathy, and a significant negative association between disease severity and Gal-9 expression was detected. In addition, the mRNA levels of Gal-9 and its ligand TIM-3 were increased in thyroid tissue from AITD patients and its expression was associated with the levels of Th1/Th12/Th17 cytokines. Immunofluorescence studies proved that intrathyroidal Gal-9 expression was confined to DCs and macrophages. Finally, in vitro functional assays showed that exogenous Gal-9 had a suppressive effect on the release of Th1/Th2/Th17 cytokines by DC/lymphocyte autologous co-cultures from both AITD patients and healthy controls.

**Conclusions:**

The altered pattern of expression of Gal-9 in peripheral blood DCs from GD patients, its correlation with disease severity as well as its ability to suppress cytokine release suggest that Gal-9 could be involved in the pathogenesis of AITD.

## Introduction

Hashimoto’s thyroiditis (HT) and Graves’ disease (GD) are the most common autoimmune thyroid diseases (AITD). In HT a Th1/Th17 response predominates, with a strong inflammatory infiltrate leading to the destruction of thyroid gland [1]. For GD, the main immunological feature is the presence of auto-antibodies directed against the thyrotropin receptor (TR-Ab), which stimulate the growth and function of thyroid follicular cells, thus leading to the development of goiter and hyperthyroidism. Graves’ ophtalmopathy (GO) is the most frequent extrathyroidal manifestation of GD, affecting up to 50% of patients. This condition is mainly due to inflammation of the orbital connective tissue and extraocular muscles, associated with an increased adipogenesis. Th1 and Th2 lymphocytes may contribute to the pathogenesis of GO at early and late stages, respectively [2]. In addition, orbital fibroblasts are capable of initiating the lymphocyte recruitment and tissue remodeling observed in GO [3].

Galectins are a family of highly-conserved glycan-binding proteins that play an important role in the innate and adaptive immune responses [4]. While an array of soluble mediators are involved in the pathogenesis of the inflammatory phenomena, different molecules promote its resolution, by inhibiting leukocyte activation and favouring the clearance of inflammatory cells [5]. Accumulating evidence indicates that galectins fall into the category of immune regulatory molecules. Thus, galectin-9 (Gal-9) down-regulates Th1 and Th17 responses and is involved in the suppression mediated by CD4+ CD25+ T regulatory (Treg) cells, mainly through interaction with the Th1-specific cell surface molecule T-cell immunoglobulin- and mucin domain- containing molecule-3 (Tim-3) [6, 7]. Several studies with experimental animal models of inflammation and autoimmunity, such as immune complex-induced arthritis, allergic asthma and type-1 diabetes, support the anti-inflammatory role of Gal-9 [7–9]. Likewise, Galectin-1 (Gal-1) acts as a negative regulator of the immune response. In this regard, in vitro and ex vivo studies have revealed that Gal-1 limits the immune response by promoting apoptosis of Th1 cells, inducing the synthesis of IL-10, and by down-regulating the release of pro-inflammatory cytokines [10, 11].

Experimental evidence indicates that in AITD there is an imbalance between the activation of Th1/Th2/Th17 effector lymphocytes and tolerogenic mechanisms, including the dysregulation of Treg cells [12, 13], and abnormalities in tolerogenic dendritic cells (DCs) [14–16]. In this regard, it is feasible that galectins may play a role in the regulation of the immunogenicity of DCs in AITD [16]. To examine the potential role of galectins in the immunopathogenesis of AITD, we analyzed the expression and function of Gal-1 and Gal-9 in DCs from patients with this condition. We found that the expression of Gal-9 was decreased in peripheral blood conventional DCs (cDCs) from GD, but not HT, patients. Functional assays showed that exogenous Gal-9 was able to inhibit the induction of Th1/Th2/Th17 responses driven by autologous monocyte-derived DCs (moDCs) from AITD patients and controls. These data suggest that Gal-9 may play a role in the pathogenesis of AITD.

## Materials and Methods

### Patients and samples

Peripheral blood samples from 36 AITD patients were studied, 11 with HT and 25 with GD. Patients with GD were recruited in a tertiary care referral centre, and a large group of them had been referred for evaluation of ophtalmopathy and/or thyroid surgery. Peripheral blood samples were also obtained from 24 healthy controls. Complete clinical and demographic data were registered in all subjects (S1 Table). At the moment of peripheral blood sampling, one HT patient was hypothyroid (FT_4_ = 0.66), two had subclinical hypothyroidism (TSH = 6.30 ±1.24 μU/mL) and eight were euthyroid (TSH = 1.77 ±1.43 μU/mL). Regarding GD patients, there was a wide distribution of TSH levels; three were hyperthyroid (FT_4_ = 2.75 ±0.65ng/dL), nine had subclinical hyperthyroidism (TSH = 0.10 ±0.12 μU/mL), 13 were euthyroid (TSH = 2.82 ±1.79 μU/mL), and in 4 patients TSH was above the upper limit of normal (TSH = 4.9 μU/ml). Thirteen patients were under anti-thyroid therapy (AT) during the study.

Ophthalmopathy was diagnosed by performing a complete eye examination by an ophthalmologist at diagnosis and at every follow-up visit, and patients were classified using the European Group on Graves' Ophtalmopathy (EUGOGO)'s activity and severity scales [17]. The clinical activity score (CAS) was calculated as the sum of the products of activity, and a cut-off point of 3 out of 7 was used to diagnose active ophtalmopathy[17]. Inactive eye disease was defined as no changes in eye status over the previous 6 months. None of the patients had received corticosteroids during the previous year. Thirteen patients had Graves’ ophthalmopathy and seven of them (54%) had active disease (S2 Table). GD patients were classified as severe (goiter grade ≥ 3, and/or active ophthalmopathy with CAS ≥ 3, n = 9) and mild (goiter grade ≤ 2, and no ophthalmopathy, n = 14). Fresh thyroid tissue was obtained from four GD patients. All these patients were euthyroid under carbimazole therapy at the time of surgery. Part of the tissue was maintained in culture medium until processing, while another fragment was frozen, embedded in OCT medium and stored at -80°C. Serum FT_4_, TSH levels, and levels of antibodies against thyroglobulin (Tg), thyroperoxidase (TPO) and TR were measured as previously described [18]. FT3 was analyzed by FT3 Access DXI 800 analyzer (Beckman Coulter Brea, CA, USA). Galectin 9 was analyzed by enzyme-linked immunosorbent assay (USCN Life Science, Hubei, China). This study was approved by the Internal Ethical Review Committee of the Hospital de la Princesa, and all patients signed a written informed consent.

### RNA isolation and real-time quantitative PCR (qRT-PCR)

Total RNA was isolated from the frozen tissue with TRIzol reagent (Invitrogen Molecular Research Center Inc. Cincinnati, OH, USA). One microgram of the total RNA was reverse transcribed using High Capacity cDNA Reverse Transcription Kit with RNAse inhibitor (Applied Biosystems, Carlsbad, CA,USA), following manufacturer’s instructions. Gal-1, Gal-9, Tim-3, IFN-γ, IL-1β, IL-12β, IL-13 and IL-17 cDNAs were amplified using the Power SYBR GREEN PCR (Applied Biosystems). Normalization was carried out following internal standards β-actin and HPRT.

### Cells

Peripheral blood mononuclear cells (PBMC) were isolated by Lymphocyte Separation Medium (Lonza, BioWhittaker, Walkersville, MD, USA) gradient centrifugation. To isolate hematopoietic mononuclear cells infiltrating the thyroid gland (TMC), thyroid specimens were minced and digested with collagenase (1.0 mg/ml; Roche Molecular Biochemicals, Mannheim, Germany) in Hank´s balanced-salt solution (BioWhittaker, Verviers, Belgium) for 1 h at 37°C. Then, cells were passed through a filter (BD Biosciences, Durham, NC, USA), and mononuclear cells were isolated by Lymphocyte Separation Medium. Finally, TMC were washed twice with PBS, and resuspended in complete RPMI 1640 culture medium (Lonza, Walkersville, MD, USA). Cell viability was always higher than 95%.

### Flow cytometry analysis

To analyze galectin surface expression on peripheral blood DCs, the following mouse anti-human monoclonal antibodies (mAb) were used: PerCP-HLA-DR, FITC-CD3, FITC-CD14, FITC-CD16, FITC-CD19, FITC-CD20, V450-CD11c (BD Biosciences) and PE-CD123 (Biolegend, San Diego, CA,USA), and goat polyclonal anti-Gal-1 or anti-Gal-9 (R&D systems, McKinley, MN,USA), followed by an AlexaFluor 647 donkey-anti-goat (DAG) (Applied Biosystems, Carlsbad, CA,USA). Gating strategy is shown in S1 Fig. To analyze Tim-3 expression, lymphocytes were stained with PE-Tim-3, APC-CD3 (Biolegend, San Diego, CA, USA), FITC-CD4, PerCP-CD8 (BD Biosciences). Thyroid DCs were stained with the following antibodies: PE-Cy7-HLA-DR (Biolegend, San Diego, CA, USA) and Pacific blue-CD11c, APC-CD123, Pacific orange-CD45 (BD Biosciences, Durham, NC, USA), and anti-Gal-1 or anti-Gal-9, followed by an AlexaFluor 647-DAG. For negative controls, we incubated cells with all primary mAb used (except for goat anti-Gal 1 and anti–Gal 9), IgG goat, and then with and then with Alexa donkey anti-goat (DAG) 647. Dead cells were excluded using 7-Amino-actinomycin D (7-AAD) (BD Biosciences, Durham, NC, USA) staining. To block Fc receptors, PBMC were incubated in an ice bath with normal human IgG (100 μg/ml; Jackson ImmunoResearch Europe Ltd.Suffolk, USA) for 30 min. Then, cells were stained with the indicated mAbs, washed with PBS, fixed in 1% paraformaldehyde and analyzed the same day with a FACSCanto flow cytometer (Becton Dickinson, Franklin Lakes, NJ, USA). The mean fluorescence intensity (MFI) was registered and data was analyzed using the FlowJo software (Ashland, OR, USA). MFI values of galectin expression were normalized according to the following ratio: MFI value in cells labelled with the anti-Gal Ab/MFI value of negative control (MFI Gal-1 or—Gal9/ MFI IgG Goat).

### Analysis of galectins in thyroid tissue by immunofluorescence

Frozen thyroid sections were fixed and permeabilized, blocked with normal human IgG, and incubated with Goat anti-human Gal-1 or-Gal-9 Abs for one hour, followed by an AlexaFluor 488 donkey-anti-goat Ab. CD11c, CD123 and CD163 mAbs (R&D systems, McKinley, MN,USA) and a rabbit-anti-CD3 (courtesy of Miguel A Alonso-Lebrero, Centro de Biología Molecular Severo Ochoa, CSIC, Madrid Spain) were combined with the proper secondary Abs coupled to Alexa 568 and a biotinylated anti-HLA-DR Ab was used with Streptavidin-647 (Applied Biosystems, Carlsbad, CA,USA). Hoechst 33342 dye was used for cell nuclei staining, and sections were analyzed in a Leica DMR immunofluorescence microscope (Leica, Wetzlar, Germany). As negative controls for galectin staining tissue slides, we incubated with all primary mAb used, except goat anti-Gal-1 or-Gal-9 Abs; followed by the secondary Abs, including the Alexa Fluor DAG 488 (S2A and S2B Fig). To quantify the percentage of dendritic cells that expressed Gal-1 or Gal-9, a minimum of 100 cells (CD11c+) or 50 cells (CD123+) per slide were analyzed.

### Functional assays

Peripheral blood lymphocytes (PBLs) isolated by gradient centrifugation using Lymphocyte Separation Medium (Lonza, BioWhittaker) were co-cultured with autologous monocyte-derived DCs (moDCs), previously generated in vitro with IL-4 (10 ng/ml) and GM-CSF (500 U/ml) and preloaded with the superantigen staphylococcal enterotoxin E (SEE) (0.1 μg/ml). Cells were cultured for 5 days in the presence or absence of human recombinant Gal-9 (hGal-9 10 μg/ml) (R&D systems, McKinley, MN, USA), where indicated lactose (50mM) was added. Then, culture supernatants were obtained and the concentrations of IFN-γ, IL-10, and IL-13 were determined using the BD CBA Human Soluble Protein Flex Set System and the FCAP Array Software Version 3.0 (BD Biosciences, Durham, NC, USA). To study the effect of hGal-9 on cell death, the co-cultured cells were stained with 7-AAD (BD Biosciences) for three assays and analyzed by flow cytometry.

### Statistical methods

Statistical analyses were carried out using GraphPad Prism (GraphPad Software, San Diego, CA, USA) and SPSS v16.0 (SPSS, Chicago, IL, USA). The association between two variables was determined with Spearman correlation analyses. Differences between groups were analyzed with Student’s t-test and one-way analysis of variance (ANOVA) with post-hoc analysis (Bonferroni's multiple comparisons test). The Mann-Whitney U and Kruskal-Wallis tests were used when data were not normally distributed.

## Results

### Expression of Gal-1 and Gal-9 by peripheral blood DCs from AITD patients

**Fig 1:**
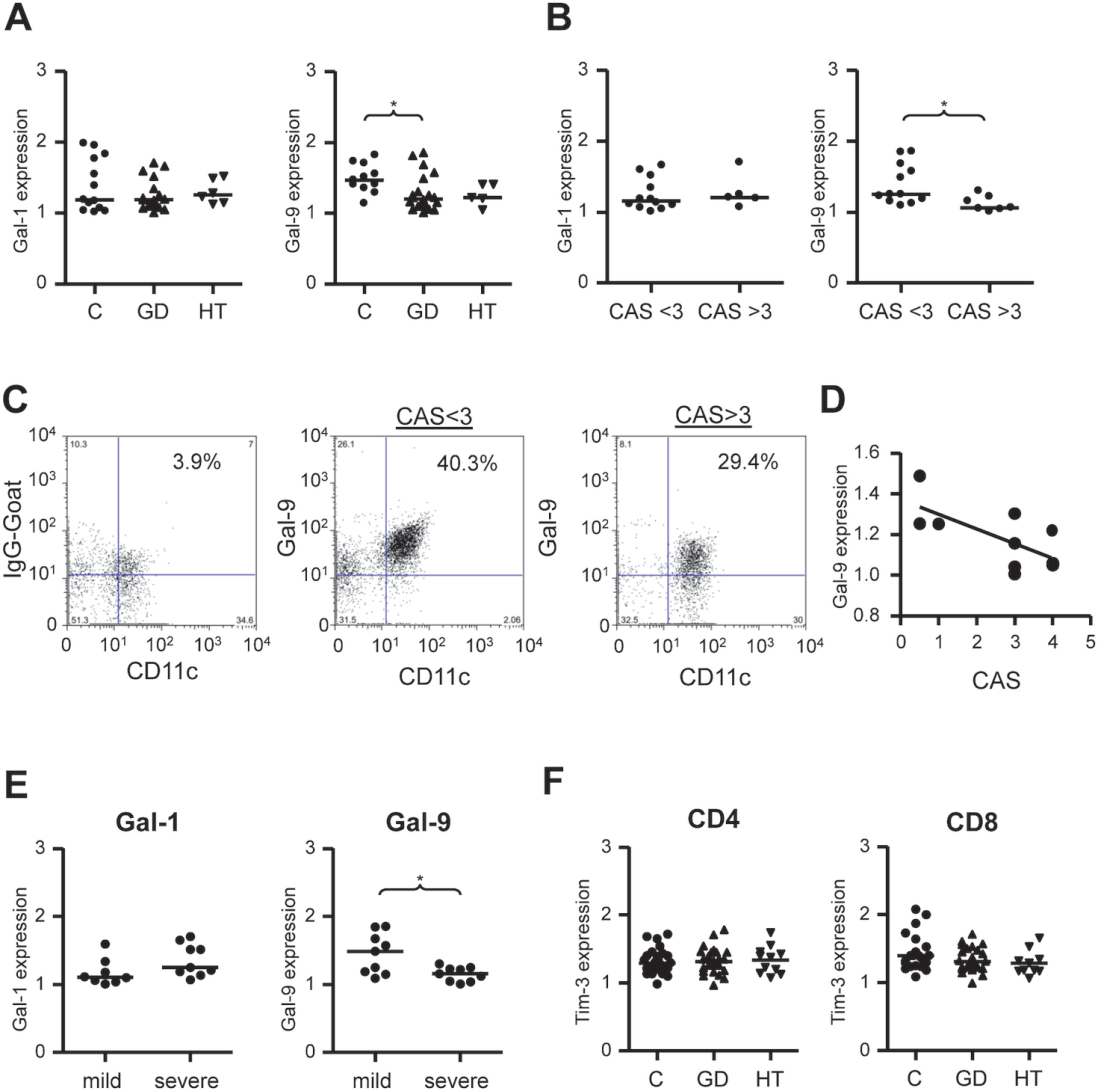
Expression of Gal-1 and Gal-9 by peripheral blood cDCs in patients with AITD. Blood samples were obtained from patients with HT, GD, and healthy controls (C), and Gal-1 and Gal-9 expression by DCs was analyzed by multiparametric flow cytometry, as described in Materials and Methods. **A**) Mean fluorescence intensity (MFI) of Gal-1 and Gal-9 expression in cDCS from GD and HT patients and healthy controls. **B)** MFI of Gal-1 and Gal-9 expression in cDCs from patients with GD, classified according to the presence or absence of active ophthalmopathy (CAS > 3 and CAS < 3 respectively). Galectins expression corresponds to normalized values (MFI Gal/MFI IgG goat) as described in Material and Methods.**C**) Representative flow cytometry dot plots of Gal-9 expression by cDCs from two GD patients, with CAS>3 and CAS<3 respectively. **D**) Linear correlation analysis of Gal-9 expression by cDCs from patients with GD and corresponding CAS. **E**) Levels of Gal-1 and Gal-9 expression by cDCs from GD patients classified according to disease severity. **F**) Expression of the Gal-9 ligand Tim-3 by CD4+ and CD8+ cells in HT, GD and healthy controls. Differences were evaluated by Kruskal-Wallis and post hoc analyses (Dunnett’s test) or the Mann-Whitney U test. *p<0.05, **p<0.01, ***p<0.001.

**Fig 2:**
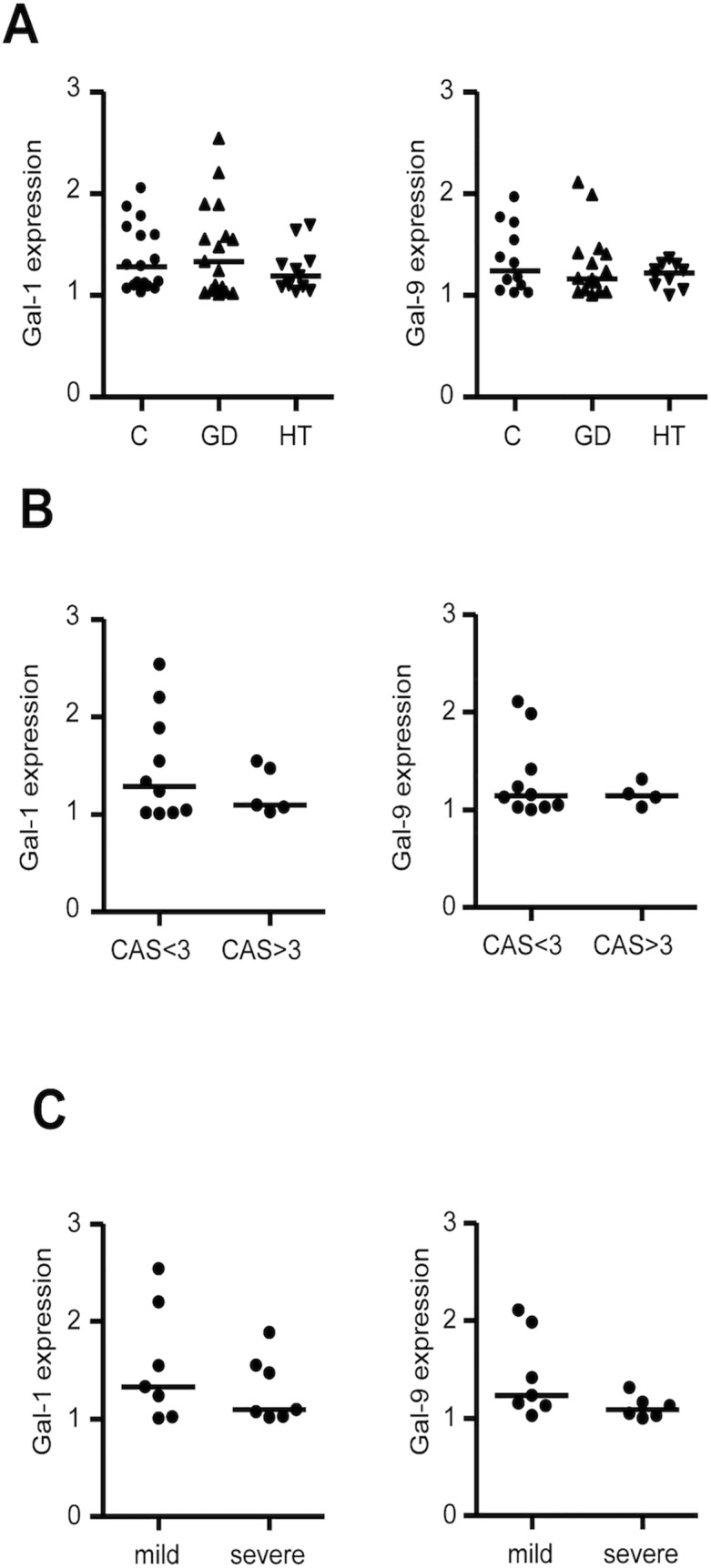
Analysis of Gal-1 and Gal-9 expression in peripheral blood pDCs from AITD patients according to disease category and activity. Peripheral blood samples were obtained from patients with HT, GD, and healthy controls, and analyzed for Gal-1 and Gal-9 expression by multiparametric flow cytometry, as described in Materials and Methods. **A**) Mean fluorescence intensity (MFI) of Gal-1 and Gal-9 expression by pDCs in patients with HT and GD and healthy controls. **B**) MFI of Gal-1 and Gal-9 expression in pDCs from patients with GD, classified according to ophthalmopathy activity. **C**) Levels of Gal-1 and Gal-9 expression in pDCs from patients with GD, classified according to disease severity. Galectins expression corresponds to normalized values (MFI Gal/ MFI IgG goat) as described in Material and Methods.

To explore the potential role of galectins in the immunopathogenesis of AITD, we first analyzed their expression in peripheral blood cDCs (CD11c+) and plasmacytoid DCs (pDCs, CD123+) from patients with HT and GD, and healthy controls by multi-parametric flow cytometry (Fig 1). There were no differences in the distribution of galectins in relation to age or gender. We found a significant decrease in the surface expression of Gal-9 on cDCs from GD patients (MFI 1.20), compared to healthy controls (MFI 1.51, p = 0.0176, Fig 1A). In contrast, although Gal-9 expression tended to be lower in HT patients compared to controls, no significant differences were observed (Fig 1A). Moreover, Gal-9 expression by cDCs was lower in the group of GD patients with active ophthalmopathy (CAS ≥ 3, MFI 1.06) compared to patients with inactive ophthalmopathy (CAS <3, MFI 1.25) (p = 0.030, Fig 1B and 1C). Accordingly, a significant inverse correlation between CAS and Gal-9 expression by cDCs was detected [17] (r = -0.679, p = 0,031, Fig 1D). There were no statistical differences in FT_4_ or TSH levels between groups with CAS < 3 and CAS> 3 (p = 0.745 and p = 0.841, respectively). In GD patients, the expression of Gal-9 was lower in those with severe clinical disease (goiter grade ≥3, and/or active ophthalmopathy with CAS ≥ 3, MFI 1.16), compared to patients with milder forms of GD (goiter grade ≤2 and CAS ≤3, MFI 1.49) (p = 0.043) (Fig 1E). In addition, no statistical difference in Gal-9 expression was found between patients who were on antithyroid treatment and those who were not (p = 0.359). No significant differences were observed for Gal-1 expression by cDCs (Fig 1A, 1B and 1E). In addition, no differences for Gal-1 and Gal-9 expression by pDCs, were found between patients and controls (Fig 2A), nor between patients with active or inactive ophtalmopathy (Fig 2B) or between patients with milder of severe clinical disease (Fig 2C). Furthermore, multivariate analysis which considered levels of expression of galectins by DCs and serum levels of FT_4_, FT3, TSH, thyroid antibodies, disease activity and anti-thyroid therapy, did not show any significant association. Likewise, no significant correlation was detected between Gal-9 expression by peripheral DCs and Gal-9 serum levels, either in patients or healthy controls (data not shown). Finally, when the expression of the Gal-9 ligand Tim-3 was analyzed in CD4+ and CD8+ T-lymphocytes, no significant differences between patients and controls were detected (Fig 1F) and Tim-3 expression was not associated with clinical disease features or severity (data not shown).

### Expression of Gal-1 and Gal-9 by thyroid DCs from patients with AITD

**Fig 3:**
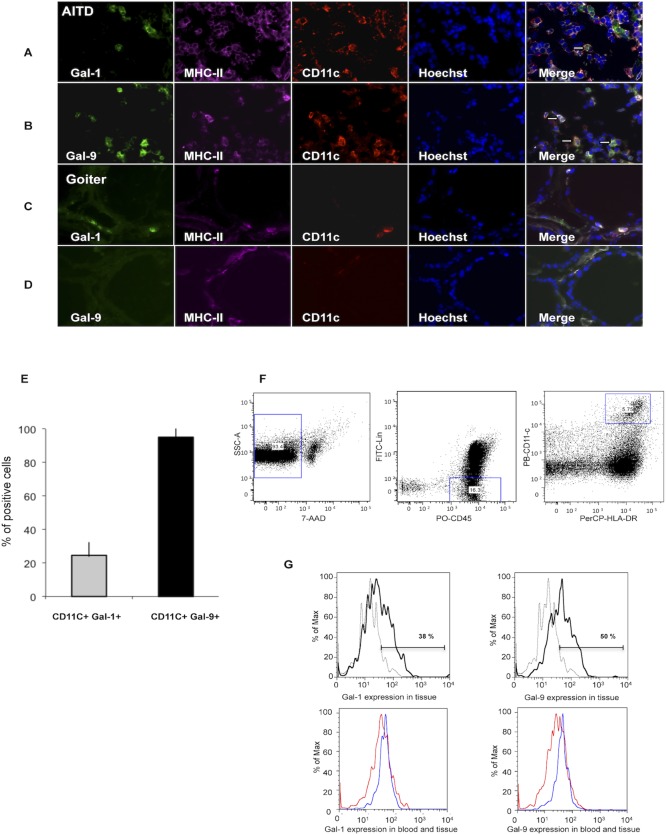
Gal-1 and Gal-9 are expressed by cDCs in thyroid tissue from patients with AITD. Triple immunofluorescence microscopy analysis of thyroid tissue from a representative AITD patient **(A,B)** and goiter**(C,D)** for the expression of Gal-1 or Gal-9 (green), HLA-DR (purple) and CD11c (red); nuclei were counterstained with Hoechst (blue); arrowheads marks some of the triple-positive cells. **E)** Percent of conventional dendritic cells expressing Gal-1 or Gal-9, a minimum of 100 cells (CD11c+) per slide were analyzed.**F, G.** Flow cytometry analysis of Gal-1 and Gal-9 expression in cDCs. **F)** Dot plot strategy to select cDCs; lineage negative cells (CD3, CD14, CD16, CD19 and CD20), CD45+, HLA-DR+, CD11c+. **G)** Representative histogram of Gal-1 and Gal-9 expression in cDCs from the thyroid gland. The percentage of Gal-1 and Gal-9 positive cells in cDCs in a representative GD patient (blue line) compared to the negative control (gray line) is shown. The apparent discrepancy between IHC and flow cytometry studies is very likely due to the different strategy to select cDCs. The Gal-1 and Gal-9 positive intrathyroidal cDCs (blue line) are compared to peripheral blood expression in the same GD patient (red line).

**Fig 4:**
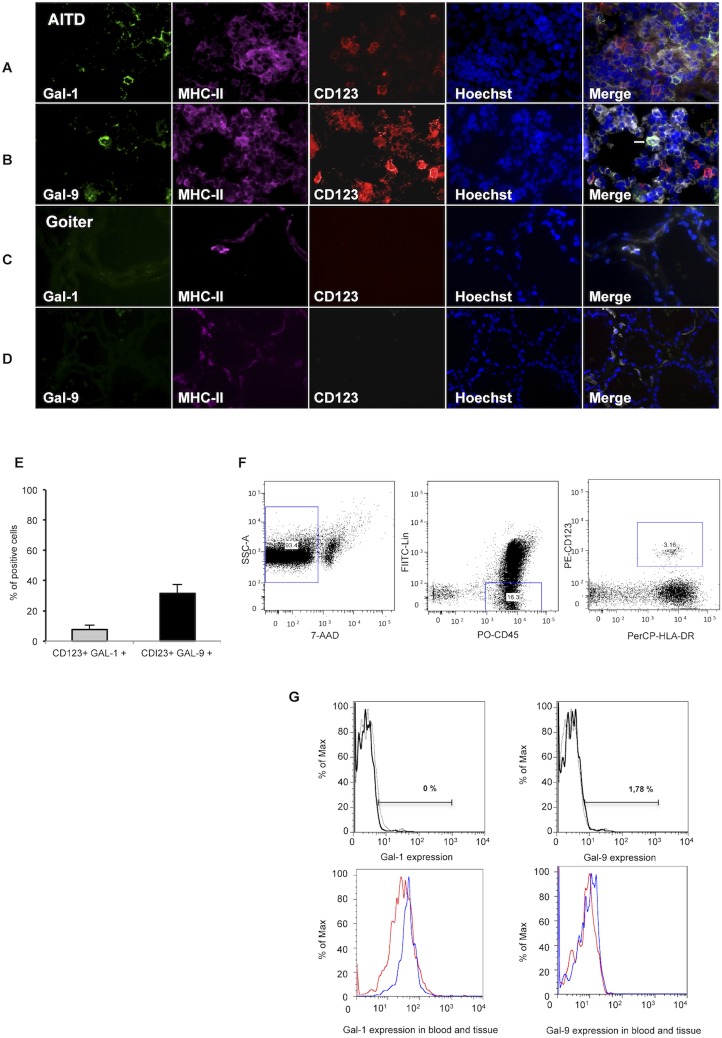
Gal-1 and Gal-9 expression by thyroid infiltrating pDCs in thyroid tissue from patients with AITD. **A-D)** Triple immunofluorescence microscopy analysis of thyroid gland sections from a representative patient with AITD (**A, B)** and goiter **(C,D)** for the expression of Gal-1 or Gal-9 (green), HLA-DR (purple) and CD123 (red); nuclei were counterstained with Hoechst (blue); arrowheads marks some of the triple-positive cells. **E)** Percent of plasmacytoid dendritic cells expressing Gal-1 or Gal-9, a minimum of 50 cells (CD123+) per slide were analyzed. **F,G.** Flow cytometry analysis of Gal-1 and Gal-9 expression in pDCs. **F)** Dot plot strategy to select pDCs; lineage negative cells (CD3, CD14, CD16, CD19 and CD20), CD45+, HLA-DR+, CD123+. **G**) Representative histogram of Gal-1 and Gal-9 expression in pDCs from the thyroid gland. The percentage of Gal-1 and Gal-9 positive cells in CD123+ intrathyroidal pDCs in a representative GD patient (blue line) compared to the negative control (gray line), is shown. The apparent discrepancy between the results between IHC and flow cytometry studies is very likely due to the different strategy to select intrathyroidal pDCs. The Gal-1 and Gal-9 positive intrathyroidal CD123+ pDCs (blue line) are compared to peripheral blood expression in the same GD patient (red line).

Triple immunofluorescence staining for MHC-II, CD11c or CD123 and galectins in thyroid tissue sections revealed expression of Gal-9 by DCs (Figs 3 and 4). Regarding Gal-1, we detected its expression in a minority of CD11c+ MHC-II+ cells (Fig 3A and 3E), while Gal-1 expression by CD123+ MHC-II+ cells was negligible (Fig 4A and 4E). Gal-9 was mainly detected in CD11c+MHC-II+ (Fig 3B and 3E) whereas few Gal-9+ CD123+MHC-II+ cells were observed (Fig 4B and 4E). When we analyzed the expression of Gal-1 and Gal-9 in other inflammatory infiltrating cells, we found no expression on CD3+ cells (S2 Fig), while a small percent of CD163+MHCII+ cells expressed Gal-9, but not Gal-1 (S3 Fig). In addition, we did not detect Gal-1 or Gal-9 expression on thyrocytes (S2 Fig and S3 Fig). Moreover, in goiter tissue sections almost no inflammatory infiltrating cells were found and no Gal-1 or Gal-9 staining was detected (Figs 3C and 3D and 4C and 4D). Expression of Gal-1 and Gal-9 in tissue dendritic cells was also analyzed by multicolor flow cytometry in CD45+ Lin-, HLA-DR+ CD11+/CD123+ cells. This analysis showed that both Gal-1 and Gal-9 were expressed in intrathyroidal cDCs (Fig 3F and 3G); however, their expression in pDCs was negligible (Fig 4F and 4G). When galectin expression by intrathyroidal cDCs and pDCs was compared with matched peripheral blood samples from four GD patients, no significant differences were detected. A representative patient is shown (Figs 3G and 4G).

### Galectin and cytokine expression in the thyroid glands from patients with AITD

**Fig 5:**
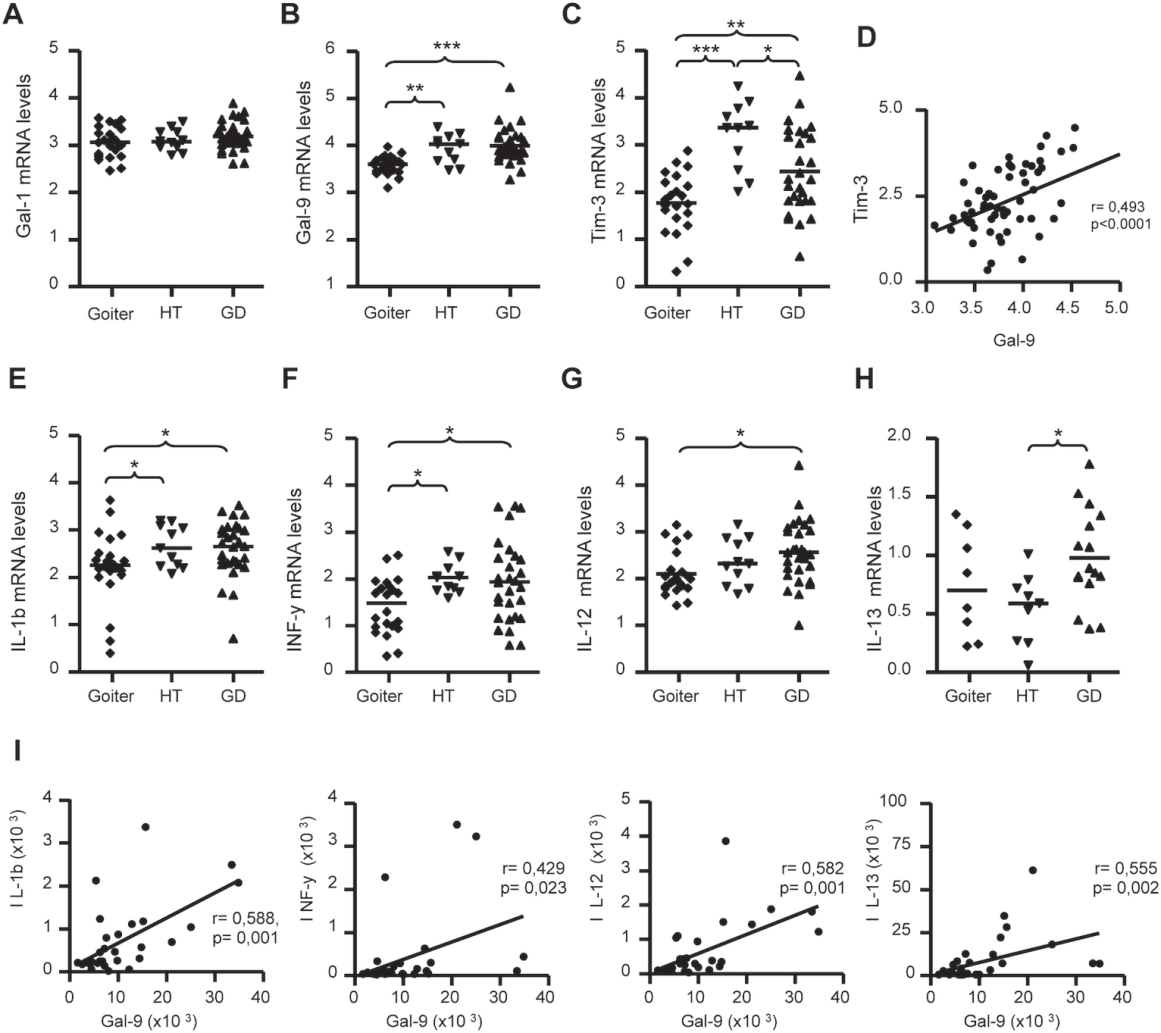
Analysis of mRNA levels of Gal-1, Gal-9 and Th1/Th2 cytokines in thyroid glands from AITD patients. Levels of mRNA of Gal-1, Gal-9, Tim-3 and the indicated Th1/Th2 cytokines were determined by qRT-PCR analysis in thyroid samples from 40 AITD patients and 22 patients with goiter. **A-C**) mRNA levels of Gal-1, Gal-9 and its ligand Tim-3 in thyroid tissue from patients with goiter, HT and GD. **D**) Correlation analysis between Tim-3 and Gal-9 mRNA levels in thyroid tissue samples from patients with GD. **E-H**) mRNA levels of the indicated Th1 and Th2 cytokines in thyroid tissue from patients with goiter, HT and GD. **I**) Correlation analysis between mRNA levels of Gal-9 and the indicated cytokines in thyroid tissue samples from patients with GD. mRNA levels were normalized using HPRT and β-actin as internal controls. Differences among groups were analysed by the ANOVA and Bonferroni tests, *p<0.05, **p<0.01, ***p<0.001. Correlation analysis was performed by Pearson or Spearman tests.

We next conducted a qRT-PCR analysis of Gal-1 and Gal-9 gene expression on thyroid samples from 40 AITD patients and 22 control samples from patients with goiter (S3 Table). We found similar levels of Gal-1 mRNA in thyroid tissue from patients with AITD and those with goiter (Fig 5A). In contrast, thyroid tissue from patients with HT and GD showed an increased expression of Gal-9 (Fig 5B, p = 0.006 and p<0.0001, compared to goiter). In addition, the expression of the Gal-9 ligand Tim-3 was significantly increased in GD and in HT compared to goiter (Fig 5C, p = 0.013 and p<0.0001, respectively), and the expression of this gene was significantly higher in HT than in GD (Fig 5C, p = 0.024). Furthermore, a significant association was detected between levels of Gal-9 and Tim3 gene expression in AITD (Fig 5D, r = 0,493, p<0.0001). Since effector Th1, Th2 and Th17 lymphocytes seem to play a key role in the pathogenesis of AITD, we analyzed the mRNA levels of Th1 (IL-1β, IFN-γ, lL-12), Th2 (IL-13) and Th17 (IL-17) cytokines in thyroid tissue. qRT-PCR analysis revealed a higher expression of IL-1β in thyroid glands from both GD and TH compared to goiters (p<0.05 in both cases, Fig 5E). Similarly, IFN-γ mRNA was increased in the thyroid tissue from both GD and TH patients (p = 0,033 and p = 0.013, Fig 5F), while IL-12mRNA levels were higher in GD thyroid tissue compared to controls (p = 0.019, Fig 5G). IL-13 mRNA concentration was higher in GD compared to HT (p = 0.048, Fig 5H). We did not detect any significant difference for IL-17 between the groups (data not shown). In patients with GD we detected a significant association between mRNA levels of IL-1β, IFN-γ, lL-12, and IL-13 and those of Gal-9 (Fig 5I). In a multivariate linear regression analysis including thyroid status, antithyroid therapy and the levels of IL-1b, IL-12, IL-13 and IFN-γ, a significant association was only found between Gal-9 and IL-12 (r = 0.975, p>0.001). No correlation with Gal-1 expression and cytokine expression was found.

### In vitro effect of galectin-9 on cytokine production

**Fig 6:**
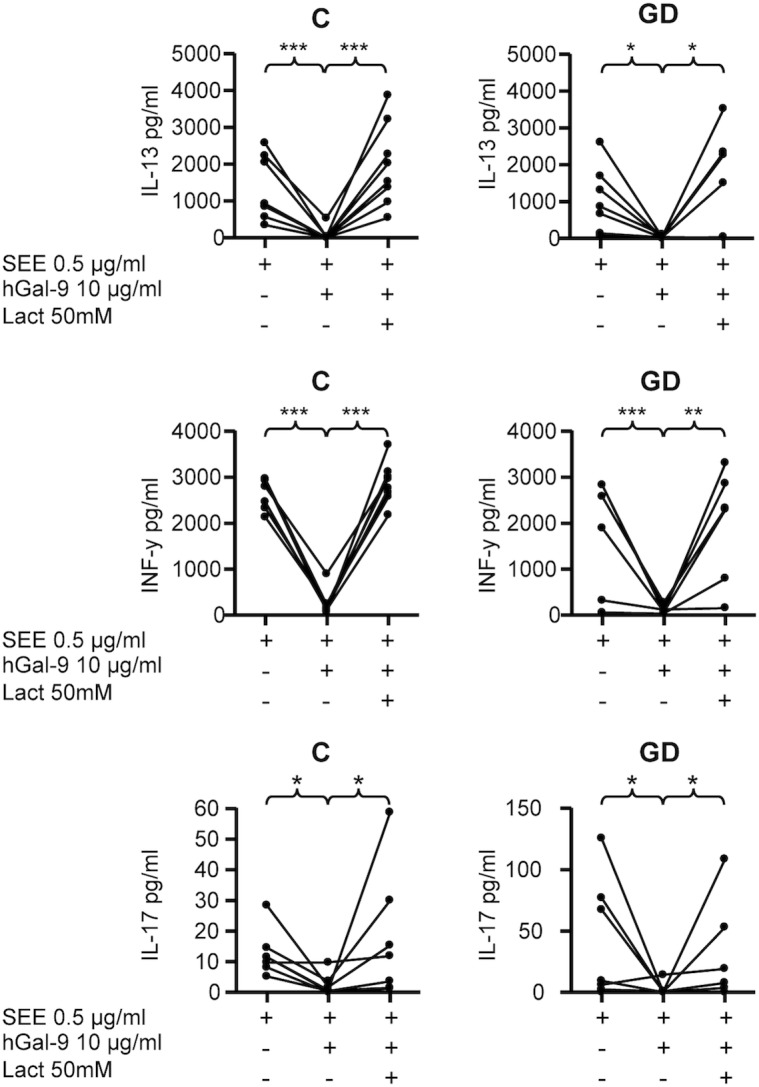
Inhibitory effect of Gal-9 on cytokine release in co-cultures of autologous moDCs with PBLs from patients with AITD and healthy controls. PBLs were co-cultured with autologous moDCs preloaded with the superantigen SEE (0,5μg/ml), in the presence or absence of 10μg/ml hGal-9 and 50 mM lactose (Lact), as stated in Materials and Methods. Then, the concentrations of the indicated cytokines in cell culture supernatants were determined by bead-based immunoassay and analysed by flow cytometry. C, healthy controls; GD, Graves’ disease.*p<0.05, **p<0.01, ***p<0.001.

Gal-1 and Gal-9 have been described as regulators of the immune responses mediated by Th1, Th2 and Th17 lymphocytes [19]. To assess this in patients with AITD, we measured IFN-γ, IL-13 and IL-17 concentrations in the supernatants of co-cultures of peripheral blood lymphocytes with autologous moDCs pulsed with SEE, in the presence or absence of hGal-9. We found that hGal-9 reduced the release of IFN-γ, IL-13 and IL-17 both in controls (p<0.01 in all cases) and patients (p<0.05 in all cases). The inhibitory effect of hGal-9 on cytokine release was similar in cells from AITD patients and controls. As expected, the addition of lactose (50mM), an inhibitor of galectin binding, abolished the regulatory effect of hGal-9 (Fig 6). In vitro cell death assays showed that the suppressive effect of Gal-9 on cytokine production was due, at least in part, to the induction of apoptosis (S4 Fig).

## Discussion

Patients with AITD show different defects in their immunoregulatory mechanisms, including an altered function of Treg lymphocytes and tolerogenic DCs, [13–16]. As galectins play an important role in regulating DC maturation and immunogenicity [19], we have assessed the expression and function of Gal-1 and Gal-9 in DCs from patients with AITD. We have found a diminished expression of Gal-9 by peripheral blood cDCs in AITD patients, mainly in those with Graves´ ophthalmopathy, and a negative association between severity of eye disease and Gal-9 expression in cDCs in GD. Galectins have been implicated in the negative regulation of the immune response, participating in the inhibition of different processes such as lymphocyte proliferation [20, 21], cellular adhesion and migration [22, 23], the induction of apoptosis, and modulation of interactions between T cells and antigen-presenting cells [24]. Accordingly, in a previous report in mice, Seki et al reported that Gal-9 deficient mice show a reduced number of Treg lymphocytes and an increased effector T cell population, leading to an enhancement of autoimmune pathology [7]. In addition, administration of recombinant Gal-9 ameliorates disease activity in various animal models of autoimmunity, including collagen induced arthritis and autoimmune diabetes [6, 7], by inhibiting the adhesion of inflammatory cells [9]. In AITD, it is feasible that the diminished expression of Gal-9 by circulating cDCs, observed in our patients, which is more evident in patients with active GO could contribute to the dysregulated recruitment of leukocytes to the orbital tissue [3]. Previous studies indicate that Gal-1 has a relevant immunosuppressive role in various experimental models of inflammation and autoimmunity [10–11], but we did not find any differences in Gal-1 expression in AITD patients.

Although we did not find any significant differences in the expression of galectins regarding thyroid function or antithyroid drug therapy, we cannot completely rule out an immunomodulatory effect of antithyroid drugs on Gal-9 expression. In this regard, it would be interesting to perform a prospective study on these patients, in order to elucidate the possible effects of hyperthyroidism, antithyroid therapy and disease progression on Gal-9 expression.

In thyroid tissue, we found increased levels of Gal-9 mRNA in thyroid samples from AITD patients that correlated with the presence of infiltrating immune cells. Accordingly, we detected Gal-9 expression in CD11c+ DCs and in CD163+ macrophages, while CD3+ T cells did not express Gal-9. In agreement with these results, Gal-9+ cDCs have been reported in the synovial tissue from patients with rheumatoid arthritis [25]. These data allow us to speculate that in chronic inflammatory conditions the expression of Gal-9 by infiltrating DCs might correspond to an immune regulatory mechanism. In addition, we found increased levels of the Gal-9 ligand Tim-3 in GD thyroid samples. In this regard the Tim-3-Gal-9 pathway is an important regulator of Th1 immunity and tolerance induction. In fact, in an experimental autoimmune encephalomyelitis model, a Th1/Th17 mediated autoimmune condition, the in vivo administration of an antibody against Tim-3 exacerbated the severity of the disease [26]. Moreover, in NOD mice, Gal-9 showed a therapeutic potential in type 1 diabetes,as it was capable of inducing apoptosis of CD4+ Tim-3+ Th1 cells [27]. In our AITD patients, although the Gal-9-Tim-3 pathway was present in the thyroid tissue, it did not seem to be sufficient to counteract the ongoing autoimmune response. Interestingly, a similar phenomenon apparently occurs with Treg lymphocytes, which accumulate in the thyroid gland of AITD patients, but seem unable to suppress the inflammatory response [13].

Since galectins can regulate the secretion of some pro- or anti-inflammatory cytokines [4], we analyzed the expression of different cytokines in AITD thyroid tissue and their relation to galectins. Our data confirmed an increased synthesis of Th1 and Th2 cytokines, including IL-1, IFN-γ, IL-12 and IL-13, in the thyroid gland from AITD patients and very interestingly a positive correlation of these cytokines with Gal-9 gene expression. We also have showed, in co-culture experiments, a reduced release of Th1, Th12 and Th17 cytokines in the presence of hGal-9. Gal-9 seems to be involved in a negative feedback loop, where IFN-γ induces Gal-9 [28], which in turn suppresses Th1 cells in vitro, thus preventing prolonged inflammation.Accordingly, in experimental autoimmune arthritis, treatment with exogenous Gal-9 significantly decreased the synthesis of the pro-inflammatory cytokines IL-17, IL-12 and IFN-γ, with induction of Treg cells within the joint [7]. In addition to the immunoregulatory effect of Gal-9 on cytokine expression, it plays an additional suppressive effect mediated, at least in part, by the induction of apoptosis. However, as stated above, in the case of AITD, it is conceivable that the increased expression of Gal-9 in thyroid tissue and the immunoregulatory mechanisms mediated by this molecule are unable to inhibit the synthesis of the pro-inflammatory cytokines and the ongoing inflammatory reaction. In the case of AITD, it is very difficult to ascertain the role of each of these immunoregulatory mechanisms for the apparent failure of Gal-9 to inhibit the ongoing autoimmune reaction. Most evidence that points to Gal-9 as a negative regulator of immune response has been obtained in animal models of inflammatory diseases, and studies in humans are scarce. Our data support a model in which Gal-9 down-regulation in human DCs may contribute to the immune process observed in AITD. The increased recognition of galectins as immunoregulatory molecules could lead to new therapeutic approaches to restore the immune equilibrium altered in AITD.

## Supporting Information

S1 Fig Gating strategy to identify Gal-9 expression in circulating primary dendritic cells. Single cell suspensions were prepared from peripheral blood and incubated with next cocktail of antibodies: FITC-conjugated anti-CD3, anti-CD14, anti-CD16, anti-CD19, anti-CD20; PerCP-conjugated anti HLA-DR, PE-conjugated anti-CD123 and Pacific Blue-conjugated anti-CD11c. Gates selected to detect the expression of galectins in cDCs and pDCs is shown.
(TIF)

S2 Fig Gal-1 and Gal-9 expression by thyroid infiltrating T-lymphocytes. A, B. Negative controls for immunofluorescence staining. **A)** Images correspond to tissue section stained only with secondary antibodies (GAM-568, Strep 647 and DAG488). **B**) Staining control for galectin expression. Tissue slides were incubated with biotinylated anti-MHC-II and mouse anti-CD11c, in the absence of anti-Gal antibodies, followed by goat antimouse (GAM)-568, Streptavidin-647 and DAG-488. **C-F**. Triple immunofluorescence microscopy analysis of thyroid tissue from an AITD patient (**C, D**) and patient with goiter**(E, F**) for the expression of Gal-1 (Green), Gal-9 (green), MHCII (purple) and CD3 (red); nuclei were counterstained with Hoechst (blue). No detectable Gal-1 or Gal-9 expression was found on thyrocytes. **G**) Percent of lymphocytes CD3+ expressing Gal-1 or Gal-9. A minimum of 100 cells (CD3+) per slide were analyzed.
(TIF)

S3 Fig Thyroid infiltrating macrophages express Gal-9, but not Gal-1 in thyroid tissue from patients with AITD. Triple immunofluorescence microscopy analysis of a thyroid from an AITD patients (**A-B**) and a goiter (**C-D**) for the expression of Gal-1 (Green), Gal-9 (green), MHCII (purple) and CD163 (red); nuclei were counterstained with Hoechst (blue). **E,F** Immunohistochemical staining for Gal-9 in AITD and in goiter. **G)** Percentage of macrophages expressing Gal-1 or Gal-9. A minimum of 100 cells (CD163+MHC+) per slide were analyzed.
(TIF)

S4 Fig hGal-9 induces cell death. Peripheral blood lymphocytes were co-cultured with autologous moDCs preloaded with the superantigen SEE, in the presence or not of hGal-9 and lactose (Lact) and cell viability was determined by staining with 7-AAD (BD Biosciences). Representative flow cytometry dot plots for each condition.
(TIF)

S1 Table Characteristics of patients and controls included in the study. (DOC)

S2 Table Goiter grade and Ophthalmopathy score in GD patients. (DOC)

S3 Table Hormone values, treatment, and intrathyroidal cytokine levels according to clinical diagnosis. (DOC)
