# Supplementary figures and images for: Graves’ Disease Is Associated with a Defective Expression of the Immune Regulatory Molecule Galectin-9 in Antigen-Presenting Dendritic Cells
Source: PLoS One. 2015 Apr 16;10(4):e0123938. doi: 10.1371/journal.pone.0123938 (PMC4399981; doi:10.1371/journal.pone.0123938)

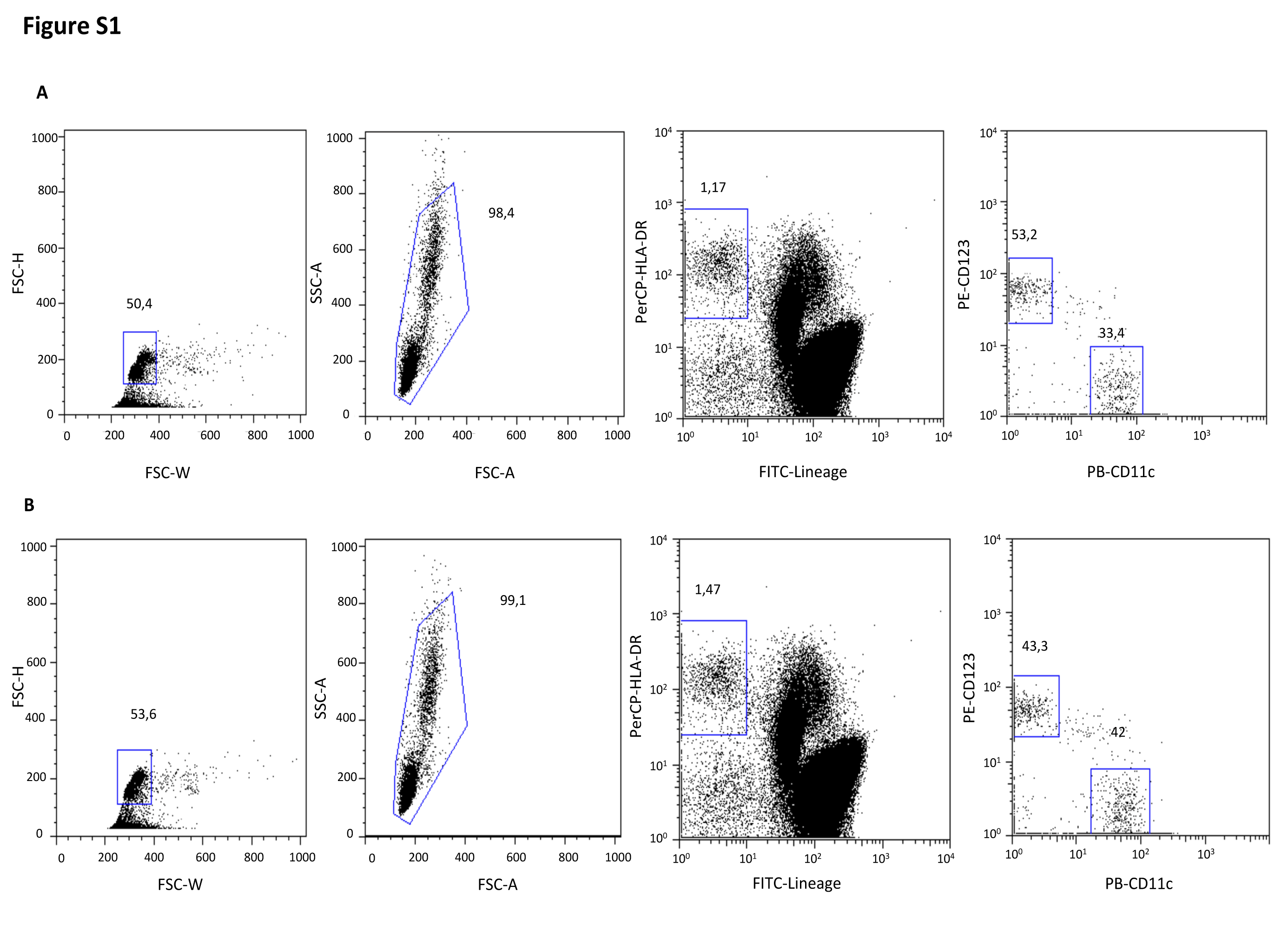

Supplement: S1 Fig — Single cell suspensions were prepared from peripheral blood and incubated with next cocktail of antibodies: FITC-conjugated anti-CD3, anti-CD14, anti-CD16, anti-CD19, anti-CD20; PerCP-conjugated anti HLA-DR, PE-conjugated anti-CD123 and Pacific Blue-conjugated anti-CD11c. Gates selected to detect the expression of galectins in cDCs and pDCs is shown. (TIF) [file pone.0123938.s001.tif]

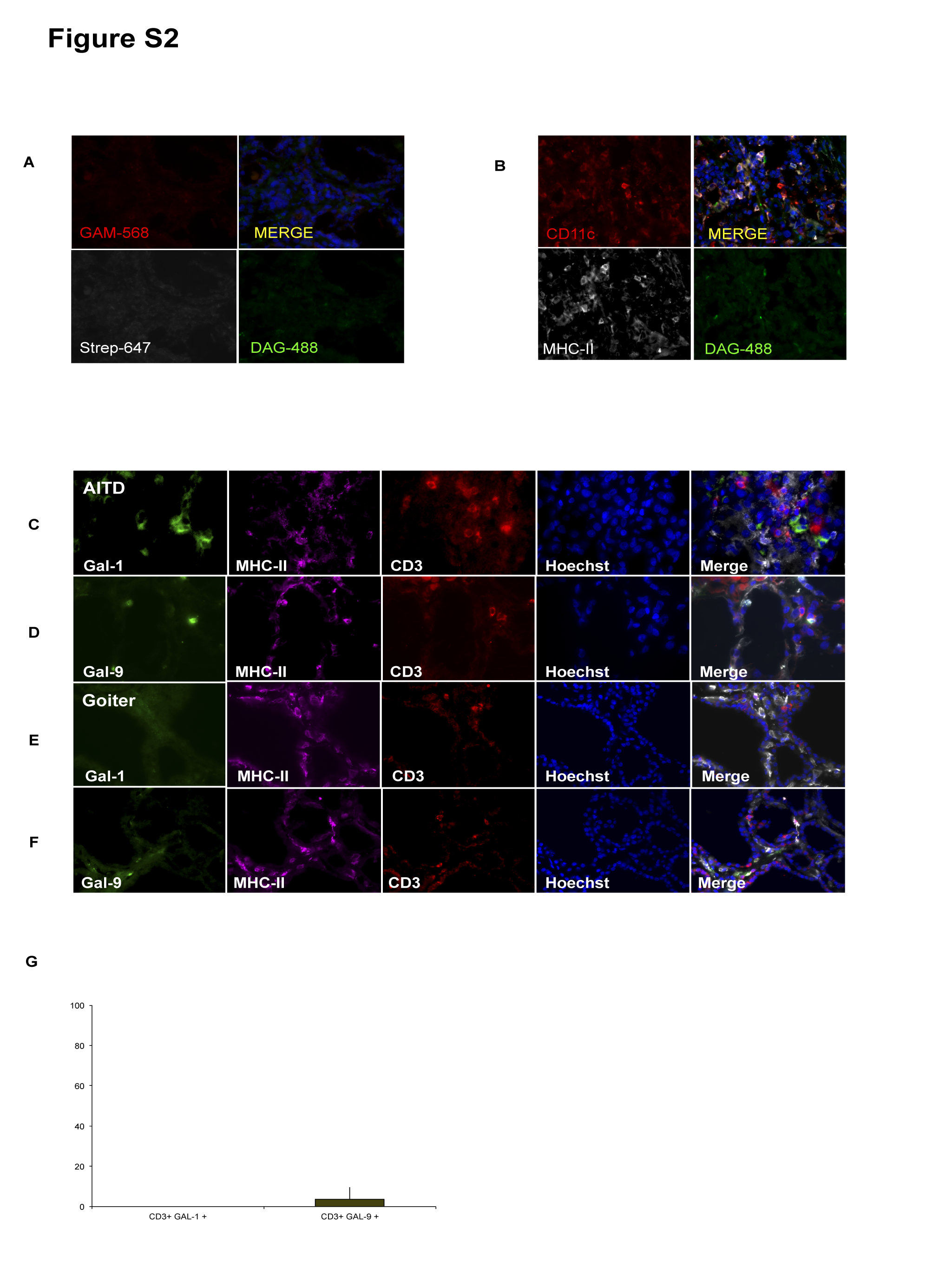

Supplement: S2 Fig — A, B. Negative controls for immunofluorescence staining. A) Images correspond to tissue section stained only with secondary antibodies (GAM-568, Strep 647 and DAG488). B) Staining control for galectin expression. Tissue slides were incubated with biotinylated anti-MHC-II and mouse anti-CD11c, in the absence of anti-Gal antibodies, followed by goat antimouse (GAM)-568, Streptavidin-647 and DAG-488. C-F. Triple immunofluorescence microscopy analysis of thyroid tissue from an AITD patient (C, D) and patient with goiter(E, F) for the expression of Gal-1 (Green), Gal-9 (green), MHCII (purple) and CD3 (red); nuclei were counterstained with Hoechst (blue). No detectable Gal-1 or Gal-9 expression was found on thyrocytes. G) Percent of lymphocytes CD3+ expressing Gal-1 or Gal-9. A minimum of 100 cells (CD3+) per slide were analyzed. (TIF) [file pone.0123938.s002.tif]

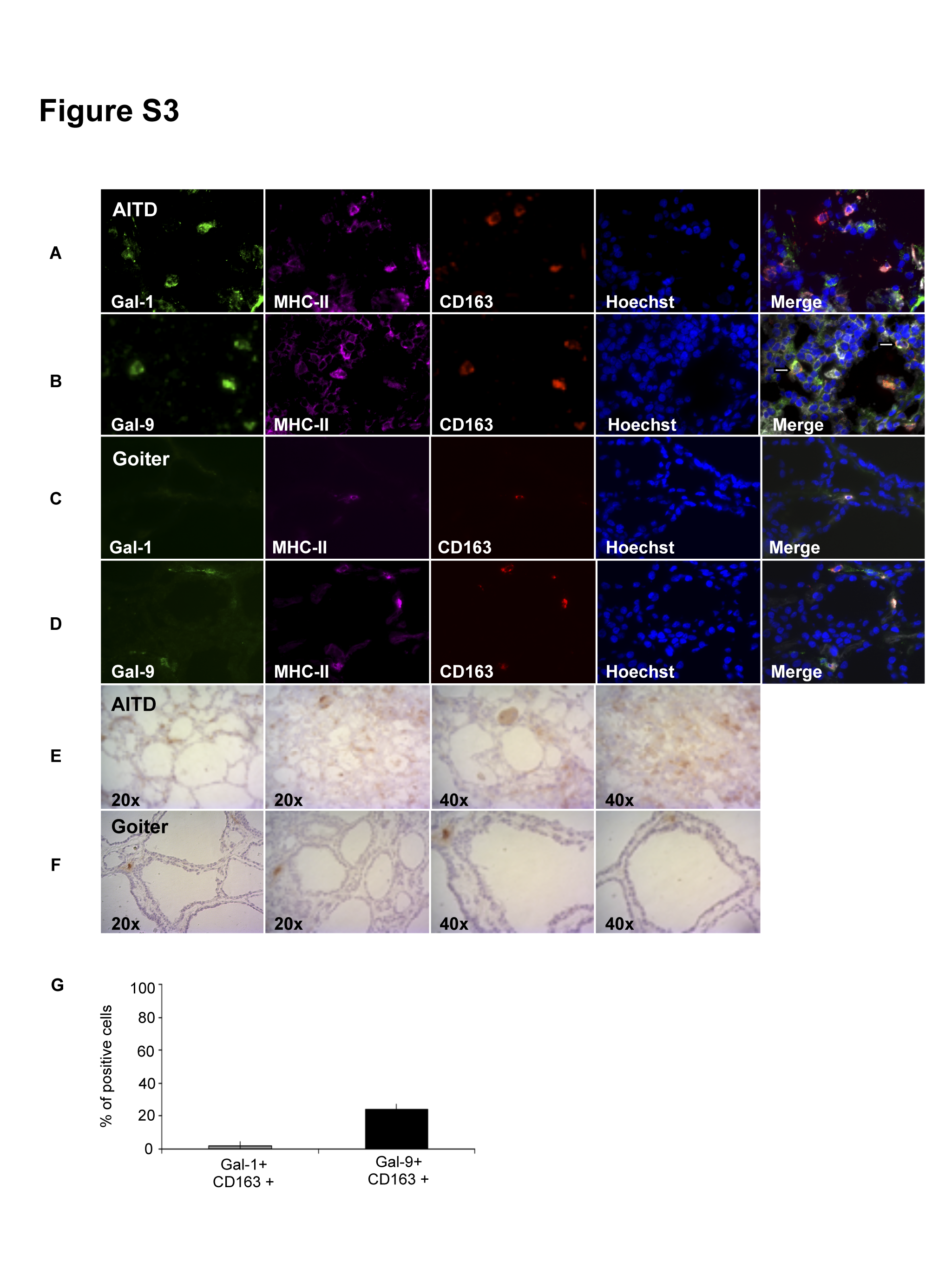

Supplement: S3 Fig — Triple immunofluorescence microscopy analysis of a thyroid from an AITD patients (A-B) and a goiter (C-D) for the expression of Gal-1 (Green), Gal-9 (green), MHCII (purple) and CD163 (red); nuclei were counterstained with Hoechst (blue). E,F Immunohistochemical staining for Gal-9 in AITD and in goiter. G) Percentage of macrophages expressing Gal-1 or Gal-9. A minimum of 100 cells (CD163+MHC+) per slide were analyzed. (TIF) [file pone.0123938.s003.tif]

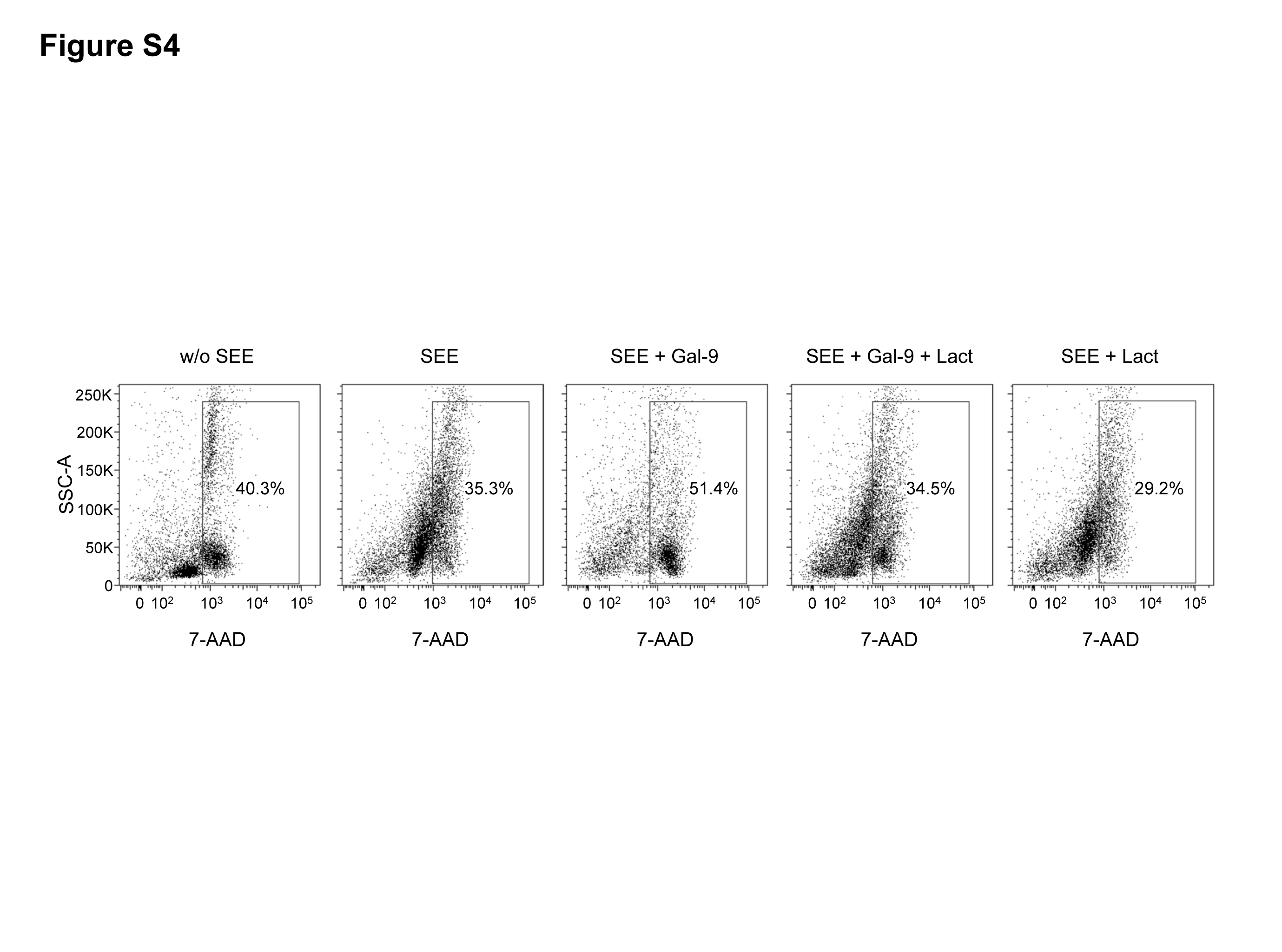

Supplement: S4 Fig — Peripheral blood lymphocytes were co-cultured with autologous moDCs preloaded with the superantigen SEE, in the presence or not of hGal-9 and lactose (Lact) and cell viability was determined by staining with 7-AAD (BD Biosciences). Representative flow cytometry dot plots for each condition. (TIF) [file pone.0123938.s004.tif]
