# Supplementary material for: Graves’ Disease Is Associated with a Defective Expression of the Immune Regulatory Molecule Galectin-9 in Antigen-Presenting Dendritic Cells
Source: PLoS One. 2015 Apr 16;10(4):e0123938. doi: 10.1371/journal.pone.0123938 (PMC4399981; doi:10.1371/journal.pone.0123938)
Supplement: S1 Table — (DOC) [file pone.0123938.s005.doc]

**Table S1. Characteristics of patients and controls included in the study**

| **Characteristics** | **All patients** | **%** | **HT patients** | **%** | **GD patients** | **%** | **Controls** | **%** |
| --- | --- | --- | --- | --- | --- | --- | --- | --- |
| **Diagnosis** |  |  |  |  |  |  |  |  |
| patients and controls | 36 | 100% | 11 | 30.60% | 25 | 69.40% | 24 | 100% |
|  |  |  |  |  |  |  |  |  |
| **Age at study entry** (years) |  |  |  |  |  |  |  |  |
| Mean ± SD | 46.0 ± 13.1 | | 50.0 ± 13.9 | | 44.7 ± 13.0 | | 41.1 ± 15.5 | |
| (minimum-maximum) | 19-67 | | 19-67 | | 23-67 | | 18-65 | |
| **Gender** |  |  |  |  |  |  |  |  |
| Male | 7 | 19.4 | 3 | 27.3 | 4 | 16 | 4 | 16.7 |
| Female | 29 | 80.6 | 8 | 72.7 | 21 | 84 | 20 | 83.3 |
| **Euthyroid^1^** |  |  |  |  |  |  |  |  |
| yes | 21 | 58.3 | 8 | 72.7 | 13 | 52 | 24 | 100% |
| no | 15 | 41.7 | 3 | 27.3 | 12 | 48 | 0 | 0 % |
| unknown |  |  |  |  |  |  |  |  |
| **Free FT4 (**ng/dL) | 1.35 ± 0.68 | | 1.49 ± 0.75 | | 1.30 ± 0.67 | | ND |  |
| **TSH (**μU/mL) | 1.88 ± 2.08 | | 2.92 ± 2.28 | | 1.45 ± 1.87 | | ND |  |
| **T3** (pg/mL)**^2^** | 3.25 ± 0.95 | | 2.82 ± 0.19 | | 3.40 ± 1.06 | | ND |  |
| **L-thyroxine** |  |  |  |  |  |  |  |  |
| yes | ND |  | 9 | 81.8 |  |  | 0 | 0 % |
| no |  |  | 2 | 18.2 | 25 | 100% | 24 | 100 % |
| **Ophtalmopathy** |  |  |  |  |  |  |  |  |
| yes | 22 | 61.1 | 1 | 9.1 | 13 | 52 | 0 | 0 % |
| no | 14 | 38.9 | 10 | 90.9 | 12 | 48 | 24 | 100 % |
| **Goiter** |  |  |  |  |  |  |  |  |
| yes | 18 | 50 | 2 | 18.2 | 16 | 64 | 0 | 0 % |
| no | 15 | 41.7 | 8 | 72.7 | 7 | 28 | 24 | 100 % |
| unknown | 3 | 8.3 | 1 | 9.1 | 2 | 8 | 0 | 0 % |
| **TPO-Ab** (U/ml)**^3^** |  |  |  |  |  |  |  |  |
| positive | 25 | 69.4 | 9 | 81.8 | 16 | 64 | ND |  |
| negative | 11 | 30.6 | 2 | 18.2 | 9 | 36 |  |  |
| unknown |  |  |  |  |  |  |  |  |
| **Tg-Ab** (U/ml)**^4^** |  |  |  |  |  |  |  |  |
| positive | 17 | 47.2 | 10 | 40 | 7 | 63.6 |  |  |
| negative | 19 | 52.8 | 15 | 60 | 4 | 36.4 | ND |  |
| unknown |  |  |  |  |  |  |  |  |
| **TR-Ab** (U/L)**^5^** |  |  |  |  |  |  |  |  |
| positive | 19 | 73.1 | 0 | 0 | 19 | 76 | ND |  |
| negative | 6 | 23.1 | 1 | 9.1 | 5 | 20 |  |  |
| unknown | 1 | 3.8 | 10 | 90.9 | 1 | 4 |  |  |

^1^Patients were considered euthyroid when TSH=0.35-4.9μU/ml and free FT4=0.70-1.48ng/dl

^2^ The reference values for T3 are 2.5 – 3.9 pg/mL

^3^ TPO-Ab=thyroid peroxidase antibody. Negative <100 U/mL (Phadia. Thermofisher. Waltham. MA)

^4^ Tg-Ab=Thyroglobulin antibody. Negative <344 U/mL (Phadia. Thermofisher. Waltham. MA)

^5^ TR-Ab=TSH-receptor antibody. Negative: < 0.5 U/L. Borderline: 0.5-0.7 U/L. Positive: > 0.7

ND= Non determined
