# Supplementary material for: Graves’ Disease Is Associated with a Defective Expression of the Immune Regulatory Molecule Galectin-9 in Antigen-Presenting Dendritic Cells
Source: PLoS One. 2015 Apr 16;10(4):e0123938. doi: 10.1371/journal.pone.0123938 (PMC4399981; doi:10.1371/journal.pone.0123938)
Supplement: S2 Table — (DOC) [file pone.0123938.s006.doc]

**Table S2. Goiter grade and Ophthalmopathy score in GD patients**

| **Characteristics** | **GD patients** | **%** |
| --- | --- | --- |
| **Goiter grade** |  |  |
| 0 | 8 | 32 |
| 1 | 4 | 16 |
| 2 | 8 | 32 |
| 3 | 3 | 12 |
| 4 | 1 | 4 |
| ND | 1 | 4 |
| **Ophthalmopathy score^1^** |  |  |
| 0 | 13 | 52 |
| 1 | 2 | 8 |
| 2 | 3 | 12 |
| 3 | 4 | 16 |
| 4 | 3 | 12 |

**^1^** Ophthalmopathy was classified using the recommendations of the European Group on Graves' Orbitopathy (EUGOGO)'s activity and severity scales (18). The clinical activity score (CAS) was calculated as the sum of the products of activity and a cut-off point of 3 out of 7 was used to diagnose activity (18).
