# Supplementary material for: Graves’ Disease Is Associated with a Defective Expression of the Immune Regulatory Molecule Galectin-9 in Antigen-Presenting Dendritic Cells
Source: PLoS One. 2015 Apr 16;10(4):e0123938. doi: 10.1371/journal.pone.0123938 (PMC4399981; doi:10.1371/journal.pone.0123938)
Supplement: S3 Table — (DOC) [file pone.0123938.s007.doc]

**Table S3. Hormone values, treatment, and intrathyroidal cytokine levels according to clinial diagnosis.**

| **Characteristics** | **All patients** | **%** | **HT patients** | **%** | **GD patients** | **%** | **Goiter patients** | **%** |
| --- | --- | --- | --- | --- | --- | --- | --- | --- |
| **Diagnosis** |  |  |  |  |  |  |  |  |
| **patients and controls** | 62 | 100% | 11 | 17.7% | 29 | 46.7% | 22 | 35.5% |
| **Free FT4 (ng/dL)** | 1.65 ± 1.35 | | 1.30 | | 2.03 ± 1.63 | | 1.08 ± 0.19 | |
| **TSH (μU/mL)** | 1.73 ± 3.85 | | 2.22 ± 1.84 | | 0.87 ± 1.88 | | 1.27 ± 0.91 | |
| **Interleukin-1 beta** | 662.5 ± 825.8 | | 692.9 ± 585.3 | | 754.4 ± 816.5 | | 527 ± 991.7 | |
| **Interleukin-5** | 194.1 ± 588.9 | | 57.3 ± 79.4 | | 378.9 ± 850.5 | | 45.5 ± 62.2 | |
| **Interleukin-10** | 881.4 ± 3928.5 | | 297.7 ± 288.4 | | 1829.2 ± 5792.0 | | 37.8 ± 32.5 | |
| **Interleukin-12** | 862.1 ± 3232.0 | | 402.3 ± 437.5 | | 1538.2 ± 4779.4 | | 252.8 ± 363.7 | |
| **Interleukin-13** | 5.6 ± 10.0 | | 3.5 ± 3.0 | | 8.3 ± 13.4 | | 3.2 ± 6.2 | |
| **Interleukin-17** | 10.7 ± 23.0 | | 6.3 ± 6.2 | | 13 ± 30.0 | | 11.4 ± 19.1 | |
| **Interleukin-23** | 192.9 ± 216.9 | | 299.5 ± 212.3 | | 190.6 ± 256.2 | | 167.7 ± 151.8 | |
| **Interferon gamma** | 278.9 ± 754.2 | | 133 ± 108.9 | | 529.9 ± 1082.1 | | 58.3 ± 81.1 | |
| **Antithyroid drug** |  |  |  |  |  |  |  |  |
| **yes** | NA |  | NA |  | 29 | 100 | NA |  |
| **no** | NA |  | NA |  | 0 | 0 | NA |  |
| **L-thyroxine** |  |  |  |  |  |  |  |  |
| **yes** | NA |  | 4 | 36.4 | NA |  | NA |  |
| **no** | NA |  | 7 | 63.6 | NA |  | NA |  |

Cytokines levels are expressed as mean ± sd (pg/ml). NA not applicable.
